# Chemistry of Sulfur-Contaminated Soil Substrate from a Former Frasch Extraction Method Sulfur Mine Leachate with Various Forms of Litter in a Controlled Experiment

**DOI:** 10.1007/s11270-018-3716-2

**Published:** 2018-02-17

**Authors:** Justyna Likus-Cieślik, Marcin Pietrzykowski, Marcin Chodak

**Affiliations:** 1 Department of Forest Ecology and Reclamation, Institute of Forest Ecology and Silviculture, Faculty of Forestry, University of Agriculture in Krakow, al. 29 Listopada 46, 31-425 Krakow, Poland; 2 Department of Environmental Management and Protection, AGH University of Science and Technology, al. Mickiewicza 30, 30-059 Krakow, Poland

**Keywords:** Frasch method, Remediation, Sulfur contamination, Organic matter

## Abstract

The impact of tree litter on soil chemistry leachate and sulfurous substrates of mine soils from former Jeziórko sulfur mine was investigated. Composites were used: soil substrate (less contaminated at mean 5090 mg kg^−1^ S or high contaminated at 42,500 mg kg^−1^ S) + birch or pine litter and control substrate (no litter). The composites were rinsed with distilled water over 12 weeks. In the obtained leachate, pH, EC, dissolved organic carbon, N, Ca, Mg, Al, and S were determined. Physicochemical parameters of the substrates and their basal respiration rate were determined. Rinsing and litter application lowered sulfur concentration in high contamination substrates. Pine litter application decreased EC and increased pH of the low-contaminated substrate. The substrate pH remained at low phytotoxic level (i.e., below 3.0), resulting in the low biological activity of the composites. Birch litter application increased leaching of N and Mg, indicating the possibility of an intensification of soil-forming processes in contaminated sites.

## Introduction

Sulfur is a common element in the environment and is indispensable for plants (Marschner 2011). However, excessive concentrations of sulfur have a negative impact on plants, damaging their root system, foliage, thinning of crowns, deformation of trees, and reduced growth (Tomlinson 1983). The effect of high sulfur content in the soil is the displacement of the alkaline Ca^2+^ and Mg^2+^ cations from the sorption complex and, consequently, acidification of the soil and increased mobility of the trace elements (Menz and Seip 2004). In ecosystems affected by increased sulfur deposition, microbial activity is reduced, followed by disturbed biogeochemical cycles and mineral nutrition (Menz and Seip 2004).

In the previous century, increased concentrations of SO_2_ in the atmosphere associated with fossil fuel combustion, wet deposition, and acid rain were reported (Zhao et al. 2003). Despite the reduction of sulfur deposition in recent decades, there are still some sulfur-contaminated soils, and in consequence, there is still a need to study the negative impact of sulfur excess on soil properties (Stern 2005; Sołek-Podwika et al. 2016). The problem of excessive sulfur concentration concerns especially open-strip lignite mining sites made of Miocene sands with high carbon content, where affects the microbiological soil properties and plants growth (Katzur and Haubold-Rosar 1996). Sulfurous and acidic soils also occur in areas with processing plants and in the former sites of mineral mining with the Frasch method (method of mining, where sulfur is melted underground using superheat to 140–160 °C water under high pressure and pumped as a slurry) (Larssen and Carmichael 2000; Liu et al. 2010; Likus-Cieślik et al. 2015, 2017). Such areas are unique research sites to assess the impact of high sulfur content on changes of other properties of the reclaimed soils in the biogeochemical sulfur cycle in the restored ecosystem.

One of the largest known sulfur deposits in the world is the bed located in the Tarnobrzeg district in southern Poland. The Frasch method sulfur mine Jeziórko occupied a site of about 2140 ha. Between 1993 and 2010, about 1179 ha were reclaimed (Likus-Cieślik et al. 2015). After end of sulfur extraction in Jeziórko sulfur mine, part of the area (705 ha) was reclaimed and afforested. Reclamation treatments consisted mainly of sulfurous horizon isolation in the top soil layer and neutralization of soil acidity with post-flotation lime (lime-containing sludge from the flotation enrichment process of native sulfur mined in Machów; the flotation lime contained on average 70.4% CaO and 0.24% Mg). Despite the reclamation measures, hotspots with high sulfur content in soils and acidification were found in mine lands (Likus-Cieślik et al. 2017). The observed soil sulfur concentrations reached up to 4% in the upper horizon (0–20 cm). This resulted in the inhibition of plant succession (Likus-Cieślik et al. 2017).

Negative properties of mine soils may be alleviated by applying various amendments during the mine soil reclamation. High sulfur content is not a common type of contamination; therefore, remediation and reclamation processes of this type of contaminated areas have been rarely described in the literature. However, problems of soil acidification is well known, e.g., in case of hard rock and coal mining sites which generate acidic soil conditions and acid mine drainage (AMD). The AMD may occur in former industrial sites contaminated with sulfur in mineral form or released as a result of weathering of pyrites. The AMD waters have a pH below 3.5 and a high concentration of iron and trace elements (Valente et al. 2013). Addition of liming materials is usually an essential first step to site remediation acidic soils (Guidelines 1996; EPA 2007). In case of pyrite-based acidity, EPA recommended to add organic soil amendments to revitalize soil, modify surface texture by adding organic matter or adding amendment with sand or clays, such as biosolids (EPA 2007). Organic residues immediately increase organic matter and nutrient contents, stimulate microbial activity, may reduce toxicity of metals, and contribute to neutralization of acid soils (Zornoza et al. 2016). Reclamation of extremely acid mine soils involved application of animal manures, composts, sewage sludge, and biochar (Zornoza et al. 2016; Dai et al. 2017). Application of litter to alleviate toxic acidity in mine soils received less attention although it is known that plant materials generally contain an excess of cations over inorganic anions (Noble et al. 1996), and the litter is relatively available at large amounts.

The paper presents the results of an experiment conducted under controlled conditions aimed at (i) determining the soil solution chemistry and the amount of sulfur leached from soil substrates from mine soils with heavy mineral sulfur contamination and (ii) simulation of the effect of organic matter application in the form of tree litter (birch and pine) on the dynamics of soil solution chemistry and soil microbial properties determined on basal respiration rate (RESP). A practical aspect of the research is the assessment of the possibility and rate of remediation of sulfur-contaminated soils.

## Material and Methods

### Experiment Design

Substrate samples for the experiment were collected in the former Jeziórko sulfur mine area (50°32′34N, 21°47′46E) from spots with diverse sulfur contamination of the soils with no vegetation cover (hotspots) (Likus-Cieślik et al. 2015). Two kinds of substrates with different sulfur concentration were used in the experiment: (i) LS—lower sulfur concentration with average S concentration (*n* = 4) 5090 mg kg^−1^ (ranging from 4471 to 5606 mg kg^−1^) and (ii) HS, with higher sulfur concentration, i.e. average (*n* = 4) 42,500 mg kg^−1^ S (ranging from 39,477 to 45,959 mg kg^−1^).

Two types of litter were used in the experiment: B—birch (*Betula pendula* Roth) and P—pine (*Pinus sylvestris* L.) litter, collected under the canopy of tree stands in managed forests not subjected to mining and with no sulfur contamination. The area of study, substrate, and litter sampling sites is characterized by foothill plains and a valley climate. The area of litter sampling was located in Southern Poland in Nowa Dęba Forest District near Jeziórko sulfur mine. The region has an average annual temperature of + 8.2 °C (− 1.6 °C in January and + 18.7 °C in July), and average annual precipitation ranges from 550 to 650 mm. Sandy soils, alluvial soils, and locally peat soils originally prevailed in the areas eventually taken over by the Jeziórko sulfur mine (Pietrzykowski et al. 2012).

**Fig. 1:**
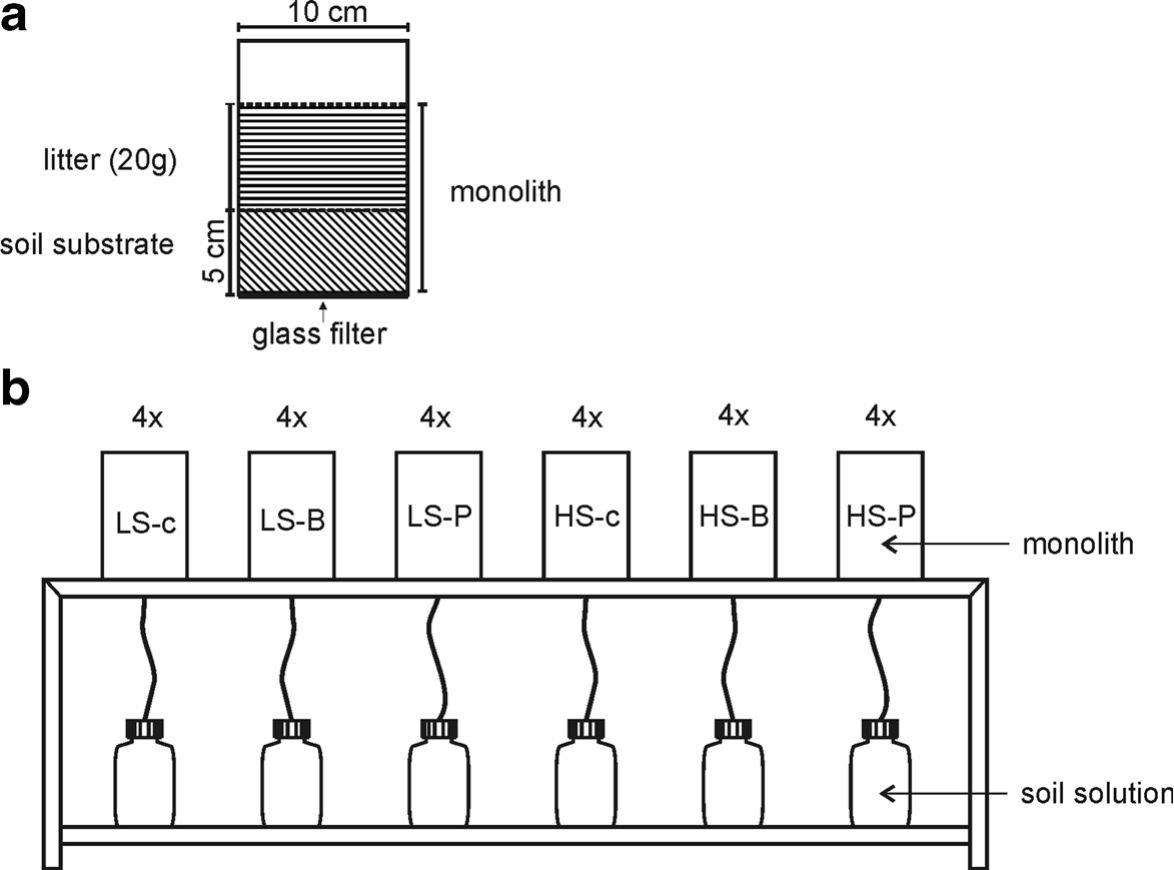
**a** Composite scheme. **b** General scheme of leaching experiment with different litterfall application (birch or pine litter) to substrate with varied sulfur concentration and control (substrate with no litter application); abbreviations: LS soil substrate with low sulfur concentration, HS soil substrate with high sulfur concentration, B common birch litter, P Scots pine litter, c control samples with no litter addition

Birch and pine litter samples collected in monoculture tree stands located on reclaimed sites of Jeziórko mine were dried in a laboratory drier (SLW 1000STD version CL/SL 1000) by 3 days in 65 °C. Litter samples were stored in the laboratory, where experiment was conducted, for 6 months, from collection to drying process. The experiment begun immediately after drying. Air-dried substrate (5 cm thickness) and litter samples (20 g) were placed in PCV cylinders (10 × 15 cm) in the following combinations (four replications each): LS-B and LS-P—substrate with low sulfur concentration + birch or pine litter, respectively; HS-B and HS-P—substrate with high sulfur concentration + birch or pine litter; and control substrate with no litter LS-c and HS-c (Fig. 1). The experiment was conducted under controlled consistent humidity and temperature (16 °C—the mean temperature of the warmest month during the vegetation season in Poland) conditions over a 12-week period. During the experiment, the cylinders were percolated with 200 ml distilled H_2_O, twice a week. Once a week, the soil solution samples were collected for laboratory analyses. To obtain a clear leachate (with no soil substrate), the bottom of the cylinder was lined with a glass microfiber filter and agrotextile.

The soil substrates and litter were analyzed at the beginning and the end (after 12 weeks) of the experiment.

### Laboratory Analyses

#### Litter and Soil Samples

The litter and soil samples were measured for pH in 1 M KCl (soil/liquid ratio 1:5 for litter and 1:2.5 for soil samples). The electric conductivity (EC) values in the soil samples were measured at 25 °C and 5:1 water/soil ratio. Organic carbon (C), total nitrogen (Nt), and total sulfur (St) contents were measured using LECO TruMac® CNS analyzer and total Ca and Mg and Al contents after digestion in a mixture of HNO_3_ and 60% HClO_4_ acid at a ratio of 3:1 (litter) and 1:3 (soil samples) by ICP-OES. Based on C and Nt content, the C/N ratio was calculated.

#### Soil Solution Analyses

The soil solutions were measured once a week for 12 weeks for pH and EC. Dissolved organic carbon (DOC) and total nitrogen content (Nt) were determined in filtered soil solution extracts with a Shimadzu TOC-VCPH total organic carbon analyzer and the concentrations of Ca, Mg, Al, and St by ICP-OES.

Based on the equation, the total sulfur load (TSL) leached from the composites in the 12-week session was calculated:

$$ {TSL}_{\left(12\kern0.5em weeks\right)}=\sum \limits_{i=1}^{12}\left({S}_i\cdot {WD}_i\right) $$

S_i_—amount of sulfur leached in a subsequent week of the experiment [mg·l^−1^]

WD_i_—amount of distilled water used to rinse the composites in a week—constant 0.2

#### Soil Biology Analyses

Soil samples after the leaching experiment were measured for basal respiration rate (RESP). To measure basal respiration, the samples (50 g d.w. for mineral substrates and 10 g d.w. for litter) were incubated at 22 °C in gas-tight jars. The incubation time was 24 h for litter and 120 h for soil substrate samples. The jars contained small beakers with 5 ml 0.2 M NaOH to trap the evolved CO_2_. After the jars were opened, 2 ml of 1 M BaCl_2_ was added to the NaOH and the excess of sodium hydroxide was titrated with 0.1 M HCl in the presence of phenolphthalein as indicator.

#### Statistical Analyses

Statistica software (StatSoft Inc. Software 2011) was used for statistical analysis. The significance of differences in average values of soil characteristics and leachate between the tested substrate variants with different levels of sulfur contamination with tree litter were tested by an ANOVA test, preceded by a Shapiro-Wilk test of normality and Levene’s test of variance homogeneity. The ANOVA test was followed by multiple pairwise comparisons using Fisher’s LSD (least significant difference) post hoc test.

In order to examine the interrelations between biological and chemical properties of the substrates, we used factor analysis (FA) based on principal component analysis. The RESP values along with the contents of SOC, N_t_, Ca, Mg, and Al, as well as pH and EC values, were standardized and used as variables to calculate the uncorrelated components accounting for the maximum amount of variance in the original data. Subsequently, the components were rotated to achieve either large or small component loadings (Varimax rotation). Interpretation of the factors was based on significant factor loading of the individual substrate on each of the factors.

## Results

### Change of Litter Parameters

At the beginning of the experiment, the litter input pH was 5.3 and 4.2 for birch and pine, respectively (Table 1). After the experiment, the pH of birch litter in the LS composite increased significantly to 5.8. The pine litter pH increased significantly in both composites, to 5.6 (LS-P) and 5.4 (HS-P) (Table 1).

**Table 1:** Changes in birch and pine litter chemical parameters during a 12-weeks experiment under controlled conditions

Properties		B	LS-B	HS-B	P	LS-P	HS-P
pH		5.3a ± 0.1	5.8b ± 0.2	5.2a ± 0.3	4.2c ± 0.0	5.6d ± 0.1	5.4ad ± 0.1
St	[mg kg^−1^]	1415a ± 29	1913 a ± 380	5236b ± 1388	877 a ± 40	1301 a ± 266	4386b ± 1215
C	[g kg^−1^]	475.87ac ± 11.72	415.03b ± 45.51	414.33b ± 23.53	499.47c ± 2.29	427.55b ± 49.63	438.90ab ± 23.12
Nt		17.20a ± 0.19	18.91b ± 2.16	18.73ab ± 1.01	6.45c ± 0.35	6.01c ± 0.80	6.44c ± 0.17
Ca		8.43a ± 0.25	14.49b ± 2.22	14.21b ± 1.92	14.64b ± 0.24	9.01ac ± 0.40	10.29c ± 0.65
Mg		0.47a ± 0.02	0.91bc ± 0.14	0.84b ± 0.10	1.01c ± 0.04	0.45a ± 0.03	0.50a ± 0.03
Al		0.11a ± 0.01	0.55b ± 0.26	0.58b ± 0.26	0.23a ± 0.02	0.25a ± 0.12	0.37ab ± 0.19
C/N		28a	22b	22b	77c	68d	71d
RESP	[μg CO_2_/g/24 h]	n.d.	1736bc ± 195	1506c ± 549	n.d.	2926a ± 370	2340ab ± 329

Mean values with the same letter are not significantly different at *p* = 0.5, 2.53 ± 0.04—mean and SD

*B* birch litter before the experiment, *LS-B* birch litter after the experiment on substrate with 5090 mg kg^−1^ S, *HS-B* birch litter after the experiment on substrate with 42,500 mg kg^−1^ S, *P* birch litter before the experiment, *LS-P* pine litter after the experiment on substrate with 5090 mg·kg^−1^ S, *HS-P* pine litter after the experiment on substrate with 42,500 mg kg^−1^ S, *n.d.* no data

The St content in the litter used in composites increased after the experiment. The highest and significant increase of St concentration was observed in the case of litter on HS substrates, i.e., from 1415 (B) to 5236 mg kg^−1^ (HS-B) and from 877 (P) to 4386 mg kg^−1^ (HS-P) (Table 1).

The Nt content in birch litter increased significantly from the initial 17.20 to 18.91 g kg^−1^ for the LS-B composite and 18.73 g kg^−1^ for the HS-B composite. For the pine litter, there was no significant change in the Nt content over the experiment time (Table 1).

In both types of litter, C content decreased significantly, resulting in narrower C/N ratios after 12 weeks of the experiment (Table 1).

The contents of Ca and Mg in birch litter increased after 12 weeks of the experiment, while for the pine litter, the opposite was the case (Table 1).

Al content at the beginning of the experiment was 0.11 mg kg^−1^ in B and 0.23 mg kg^−1^ in P. After 12 weeks of experiment, Al content in B litter increased significantly (to 0.55 mg kg^−1^ in LS-B and 0.58 in HS-B; Table 1).

After 12 weeks of the experiment, higher RESP values were measured for the pine litter (2926 and 2340 μg CO_2_ g^−1^ 24 h^−1^, for litter on LS and HS substrate, respectively) than for the birch litter (1736 and 1506 μg CO_2_ g^−1^ 24 h^−1^, for litter on LS and HS substrate, respectively). There was a tendency for higher respiration values in the case of the litter on the LS substrate than on the HS substrate; however, the difference was significant only at *p* = 0.09.

### Changes of Mineral Soil Substrate Parameters

The deacidification effect was obtained by pine litter application, but only in the case of LS substrates (Table 2). In all cases, the pH of the substrates after the experiment was still phytotoxic, i.e., below 3.0.

**Table 2:** Chemical parameter changes of soil substrates during a 12-weeks experiment under controlled conditions

Properties		LS	LS-c	LS-B	LS-P	HS	HS-c	HS-B	HS-P
pH		2.1a ± 0.0	2.6bc ± 0.0	2.5bd ± 0.1	2.9e ± 0.3	2.5bc ± 0.0	2.6c ± 0.0	2.3d ± 0.0	2.5bc ± 0.1
EC	[mS cm^−1^]	1.87a ± 0.057	0.39b ± 0.032	0.59c ± 0.11	0.23d ± 0.23	2.59e ± 0.053	2.53e ± 0.075	2.98f ± 0.123	2.58e ± 0.047
St	[mg kg^−1^]	5090a ± 483	6447a ± 397	5947a ± 1319	6178a ± 1870	42521b ± 2670	35634c ± 9568	33247c ± 4033	34157c ± 4291
SOC	[g kg^−1^]	3.22a ± 0.09	2.89a ± 0.32	2.97a ± 0.16	3.09a ± 0.20	7.78b ± 0.13	7.23b ± 0.34	7.80b ± 1.14	7.20b ± 0.44
Nt		0.03a ± 0.03	0.02a ± 0.01	0.03a ± 0.01	0.02a ± 0.00	0.16b ± 0.02	0.19bc ± 0.04	0.22c ± 0.04	0.18b ± 0.01
Ca		7.06a ± 0.50	0.07b ± 0.05	0.06b ± 0.00	0.38b ± 0.36	11.68c ± 1.56	4.21de ± 2.25	2.95d ± 0.73	5.99de ± 2.23
Mg		0.09ac ± 0.01	0.04b ± 0.01	0.04b ± 0.00	0.05b ± 0.00	0.11a ± 0.00	0.08c ± 0.04	0.11a ± 0.01	0.11a ± 0.01
Al		0.97ab ± 0.09	0.37c ± 0.25	0.43c ± 0.03	0.49c 0.04	1.51e ± 0.00	0.77ad ± 0.44	1.02ab ± 0.18	1.25be ± 0.17
RESP	[μg CO_2_/g/24 h]	n.d.	2.49a ± 0.48	2.75a ± 1.38	1.91a ± 0.71	n.d.	2.40a ± 0.57	0.99a ± 0.48	2.24a ± 0.59

Mean values with the same letter are not significantly different at *p* = 0.5, 2.53 ± 0.04—mean and SD

*LS* soil substrate with 5090 mg kg^−1^ S before the experiment, *LS-c* control sample of soil substrate with 5090 mg kg^−1^ S after the experiment, *LS-B* birch litter after the experiment on substrate with 5090 mg kg^−1^ S, *LS-P* pine litter after the experiment on substrate with 5090 mg kg^−1^ S, *HS* soil substrate with 42,500 mg kg^−1^ S before the experiment, *HS-c* control sample of soil substrate with 42,500 mg kg^−1^ S after the experiment, *HS-B* birch litter after the experiment on substrate with 42,500 mg kg^−1^ S, *HS-P* pine litter after the experiment on substrate with 42,500 mg kg^−1^ S, n.d. no data

The EC values in LS substrates decreased after the experiment from 1.87 to 0.39 mS cm^−1^ in the substrates with no litter added. The decrease in EC was even larger when the pine litter was applied while the birch litter application had no significant effect (Table 2). In the case of HS substrates, the EC vales were consistently high (above 2.5 mS cm^−1^) and did not significantly decrease after 12 weeks of rinsing, and when birch litter was added even increased significantly to 2.98 mS cm^−1^ (Table 2).

**Fig. 2:**
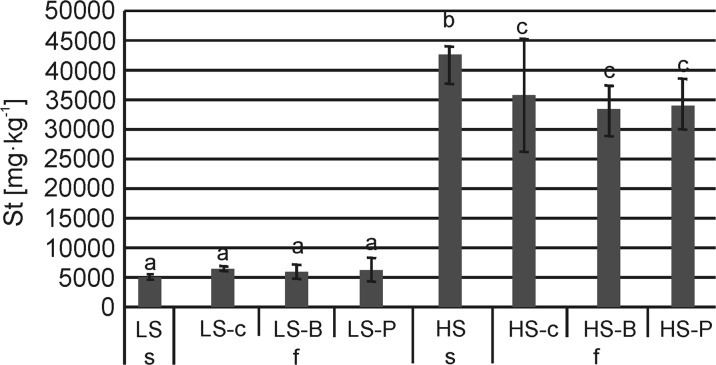
Sulfur content (St) in the substrate at the beginning and at the end of the experiment under controlled conditions; Explanation: letters (a, b) specify the significant differences between the mean values of composite at the beginning and the end of the experiment at *p* = 0.05; abbreviations: LS soil substrate with low sulfur concentration, HS soil substrate with high sulfur concentration, B common birch litter, P Scots pine litter, **c** control samples with no litter addition

The St content in the LS substrate did not decrease significantly either in the control or in the composites with litter (Fig. 2). On the contrary, in the case of HS substrates, leaching significantly reduced St concentration from 42,521 to 35,634 mg kg^−1^ (HS-c), 33,247 mg kg^−1^ (HS-B), and 34,157 mg kg^−1^ (HS-P). However, the effect of litter application was not significant (Fig. 2).

SOC content was significantly higher in HS substrates than in LS, whereas in both substrate variants, it remained at similar levels throughout the experiment (i.e., 2.89–3.22 g kg^−1^ in LS substrates; 7.20–7.80 g kg^−1^ in HS substrates) (Table 2).

The Nt content in LS substrates did not change after the experiment either in the control sample or in the litter composites (Table 2). For the HS substrates, there was a tendency for higher Nt content after the experiment; however, the observed increase was significant only for HS-B composite (Table 2).

During each experiment, Ca leached intensively from the substrates. After 12 weeks of rinsing, the Ca content decreased several-fold from initial 7.06 to 0.07 g kg^−1^ in LS-c, 0.06 g kg^−1^ in LS-B, and 0.38 g kg^−1^ in LS-P (Table 2). Similarly for the HS substrates, the Ca content also decreased several-fold from 11.68 g kg^−1^ before the experiment to 4.21 g kg^−1^ in HS-c, 2.95 g kg^−1^ in HS-B, and 5.99 g kg^−1^ in HS-P (Table 2).

Mg was not leached from substrates as intensively as Ca. For the LS substrate, the Mg content after 12 weeks decreased in all the investigated combinations from the initial concentration of 0.09 g kg^−1^ to 0.04 (LS-c, LS-B) and 0.05 g kg^−1^ (LS-P). For the HS substrates, the Mg content decreased from 0.11 to 0.08 g kg^−1^ only in the control samples. In the HS-B and HS-P composites, there was no change in Mg content (Table 2).

Initial content of Al was significantly higher in the HS substrate (1.51 g kg^−1^) than in the LS substrate (0.97 g kg^−1^). After 12 weeks of the experiment, the Al contents decreased in all composite samples (the LS samples 0.39–0.49 g kg^−1^; the HS samples 0.77–1.25 g kg^−1^). This decrease was somewhat more pronounced in the control samples than in soils with litter addition (Table 2) but the differences were not statistically significant.

The RESP values were extremely low for both substrates and varied from 0.99 to 2.49 μg CO_2_ g^−1^ 24 h^−1^ (Table 2).

**Fig. 3:**
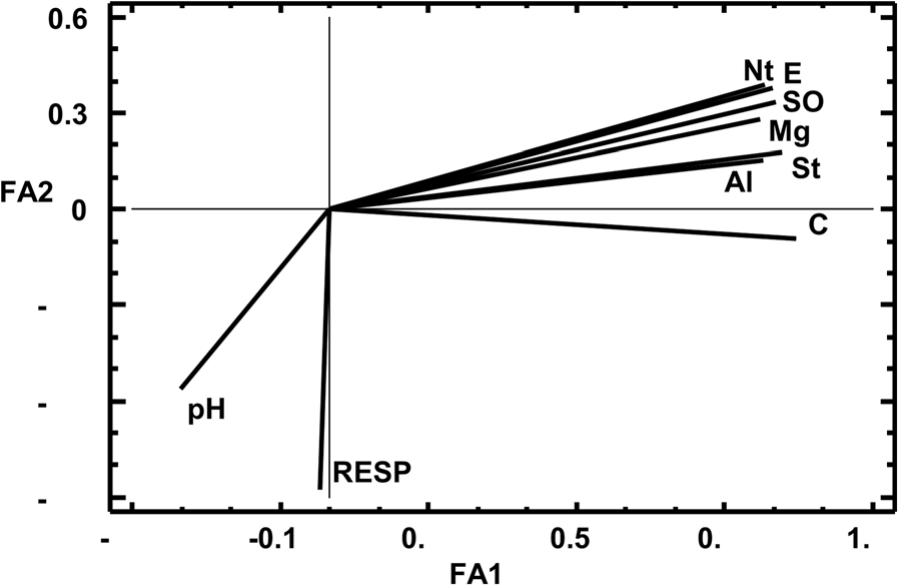
Factor analysis (FA) loading plot for different microbial (RESP) and chemical (SOC, Nt, St, Mg, Ca, Al, pH, and EC) properties of the LS and HS substrates after 12 weeks of the leaching experiment

The factor analysis with RESP and chemical properties yielded two factors that explained 70.2% (FA1) and 11.8% (FA2) of the variance, respectively (Fig. 3). The largest loadings to the first factor were from all chemical properties (loadings 0.87–0.95) except pH (− 0.30). The RESP value had only marginal loading to FA1 (− 0.02) but a large one to FA2 (− 0.87). The FA2 was related also to pH (− 0.56), but the other chemical properties had smaller loadings to FA2 (− 0.09 to 0.38).

### Dynamics of Soil Dissolution under Leaching

**Fig. 4:**
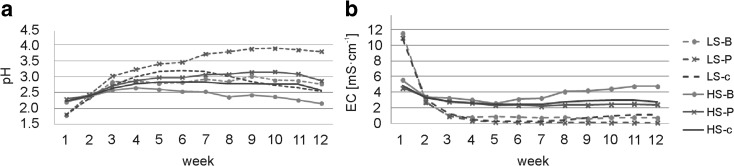
Changes in pH (**a**) and EC (**b**) of the solutions during the experiment under controlled conditions; abbreviations: LS soil substrate with low sulfur concentration, HS soil substrate with high sulfur concentration, B common birch litter, P Scots pine litter, c control samples with no litter addition

In the first week of the experiment, the solutions in both substrate variants were very acidic (pH = 1.8 for all LS variants and pH = 2.2–2.3 for HS variants) (Fig. 4a). During the experiment, all of the variants showed initial increase followed by pH stabilization; however, higher pH change dynamics was found in solutions from LS substrates (Fig. 4a). In solutions from HS-B leachate, no significant changes were observed between the pH at the beginning and at the end of the experiment (Table 3). The pH of the remaining solutions at the end of experiment increased and amounted from 2.5 (LS-c) to 3.8 (LS-P) and from 2.6 (HS-c) to 2.9 (HS-P) (Fig. 4a). After 12 weeks of the experiment, higher pHs were obtained from solutions from leachate of composites containing pine litter and lower in ones containing birch litter (Table 3).

**Table 3:** Mean values and standard deviations for pH, EC, content of dissolved organic carbon (DOC), and the concentrations of St, Nt, Ca, Mg and Al in leachates at the beginning and after 12 weeks of leaching through soil substrates and composites in controlled conditions

		LS-c	LS-B	LS-P	HS-c	HS-B	HS-P
pH	s	1.8b§ ± 0.04	1.8b ± 0.02	1.8b ± 0.10	2.2b ± 0.03	2.2 ± 0.10	2.3b ± 0.10
	f	2.5a ± 0.08	2.8a ± 0.12	3.8a ± 0.26	2.6a ± 0.18	2.2 ± 0.22	2.9a ± 0.26
EC [mS cm^−1^]	s	10.80a ± 1.38	11.48a ± 0.42	10.91a ± 3.07	4.85a ± 0.29	5.52 ± 0.77	4.51a ± 0.49
	f	1.12b ± 0.32	0.71b ± 0.23	0.10b ± 0.03	2.74b ± 0.34	4.75 ± 1.00	2.40b ± 0.36
St [mg kg^−1^]	s	1570.8a ± 156.5	1585.9a ± 100.1	1583.3a ± 428.6	814.3a ± 31.4	841.4 ± 73.6	741.0a ± 74.4
	f	71.0b ± 20.3	53.7b ± 17.2	9.6b ± 4.6	625.7b ± 36.6	725.4 ± 63.6	588.0b ± 24.0
DOC [mg l^−1^]	s	82.84a ± 8.95	92.97a ± 9.38	117.13a ± 18.80	101.41a ± 9.54	127.00a ± 33.11	110.37a ± 15.38
	f	5.08b ± 0.33	20.47b ± 3.17	37.20b ± 10.72	9.78b ± 1.65	32.00b ± 4.03	29.56b ± 2.48
Nt [mg l^−1^]	s	14.65a ± 1.97	18.05a ± 1.02	16.42a ± 4.11	5.72a ± 0.31	7.68a ± 1.55	6.54a ± 0.69
	f	0.53b ± 0.04	1.46b ± 0.17	1.17b ± 0.33	0.43b ± 0.02	2.58b ± 0.67	0.82b ± 0.05
Ca [mg l^−1^]	s	474.41a ± 39.07	445.30a ± 26.10	442.78a ± 33.14	550.23a ± 17.79	552.79a ± 14.18	553.38 ± 50.04
	f	5.84b ± 1.28	13.66b ± 4.08	5.20b ± 6.11	467.27b ± 24.03	494.78b ± 21.99	495.56 ± 17.26
Mg [mg l^−1^]	s	2.21a ± 1.73	7.85a ± 1.48	8.01a ± 3.14	0.01c ± 0.00	1.62ad ± 0.96	0.85d ± 1.70
	f	0.01b ± 0.00	0.68b ± 0.12	0.07b ± 0.14	0.01c ± 0.03	2.24a ± 0.49	0.48d ± 0.09
Al [mg l^−1^]	s	73.24a ± 12.42	61.17a ± 6.95	60.68a ± 25.23	42.95a ± 6.32	32.42a ± 10.98	21.66a ± 9.22
	f	0.22b ± 0.16	0.05b ± 0.04	0.06b ± 0.04	0.74b ± 0.41	1.77b ± 0.47	0.21b ± 0.14

Different letters indicate significant differences in the measured values at the beginning and at the end of the experiment (*p* = 0.05, pairwise *t* test)

*s* at the beginning of experiment, *f* after 12 weeks

In all the variants, the highest EC values were observed at the beginning of the experiment (weeks 1 and 2), indicating the most intensive soil leaching during this period (Fig. 4b). At the beginning of the experiment, the LS substrates had higher EC values than HS substrates indicative of faster rate of salt leaching (Fig. 4b). However, at the end of the experiment, the leachates from HS substrates had significantly higher EC values than those of LS substrates (Table 3). Addition of birch litter stimulated salt leaching from HS substrates but had no effect on LS substrates.

**Fig. 5:**
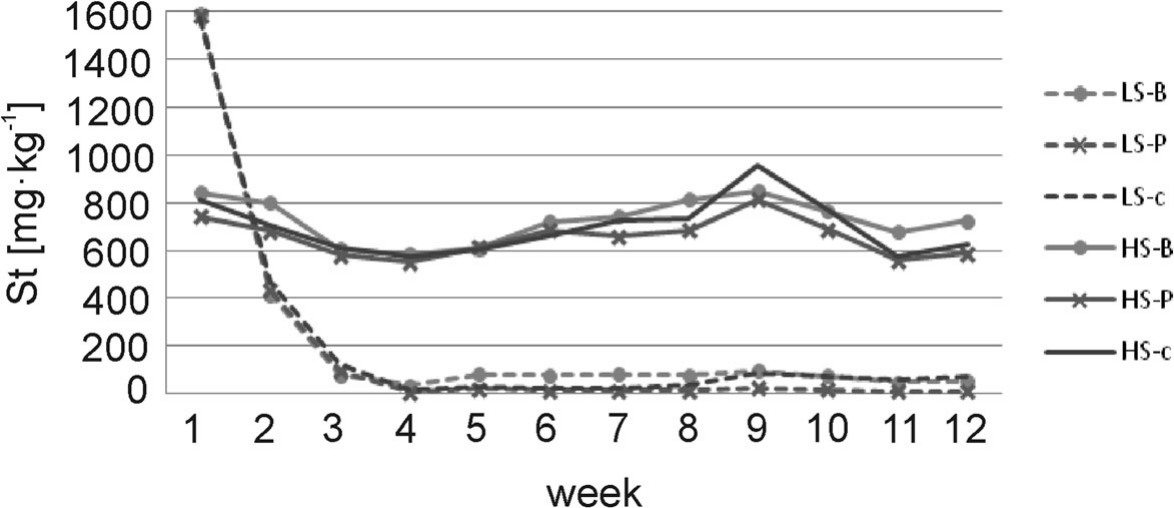
Changes in sulfur content (St) in solutions during the experiment under controlled conditions; abbreviations: LS soil substrate with low sulfur concentration, HS soil substrate with high sulfur concentration, B common birch litter, P Scots pine litter, c control samples with no litter addition

At the beginning of the experiment (week 1), significantly higher St concentrations were measured in the leachates obtained from LS substrates (Fig. 5 and Table 3). Dynamic leaching of St was observed in these solutions, which lasted up to the third week of the experiment and then stabilized at very low values (Fig. 5). After 12 weeks, the concentrations of St in the solutions from the LS substrates were significantly lower than at the beginning (Table 3). Unexpectedly, the St concentrations in solutions obtained from composites containing HS substrate were initially (week 1) lower compared to St concentration in solutions leached from LS substrates (HS-c 814.3 mg l^−1^, HS-B 841.4 mg l^−1^, HS-P 741.0 mg l^−1^). However, contrary to the LS substrates, the St leaching from HS substrates remained relatively constant throughout the experiment (Fig. 5). Only after 12 weeks, slightly lower St concentrations were measured in the LS-c and HS-P composites, while no change was found for the HS-B (Table 3).

**Fig. 6:**
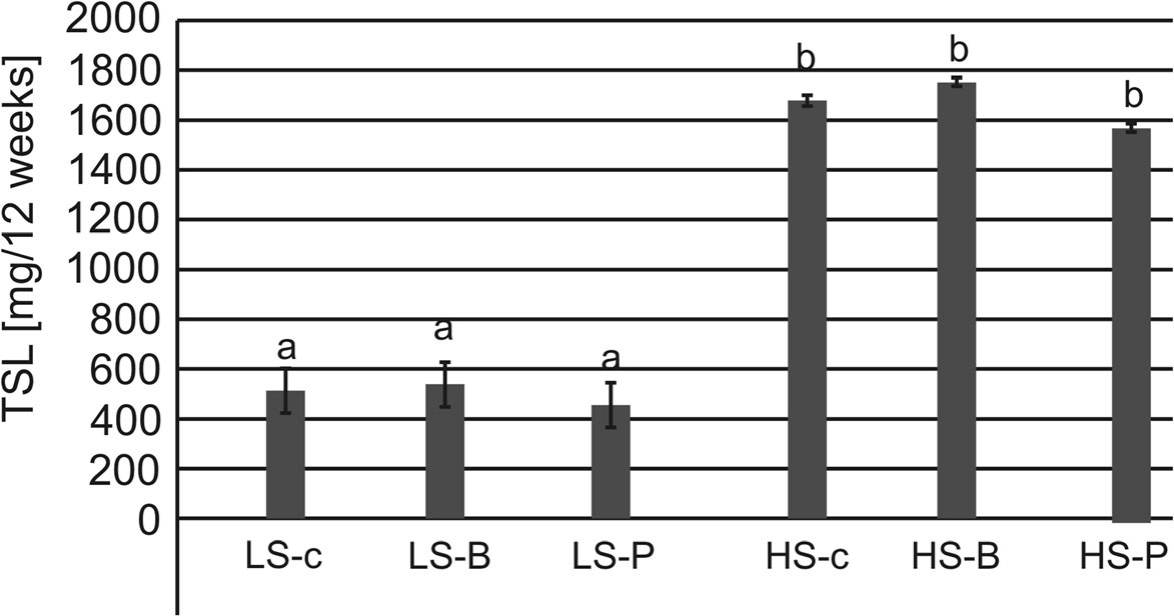
Load of sulfur leached from substrates in various combinations of composites used in the experiment. Explanations: letters (a, b) specify the significant difference between the mean values of composite at the beginning and the end of the experiment at *p* = 0.05; abbreviations: LS soil substrate with low sulfur concentration, HS soil substrate with high sulfur concentration, B common birch litter, P Scots pine litter, c control samples with no litter addition

Total sulfur load (TSL) leached from the LS substrates containing litter ranged from 449.0 (LS-P) to 541.3 mg (LS-B) and that from HS substrates was significantly higher, i.e., 1568.4 (HS-P) up to 1745.2 mg (HS-B); however, there was no effect of litter application on the TSL values after 12 weeks of leaching (Fig. 6).

**Fig. 7:**
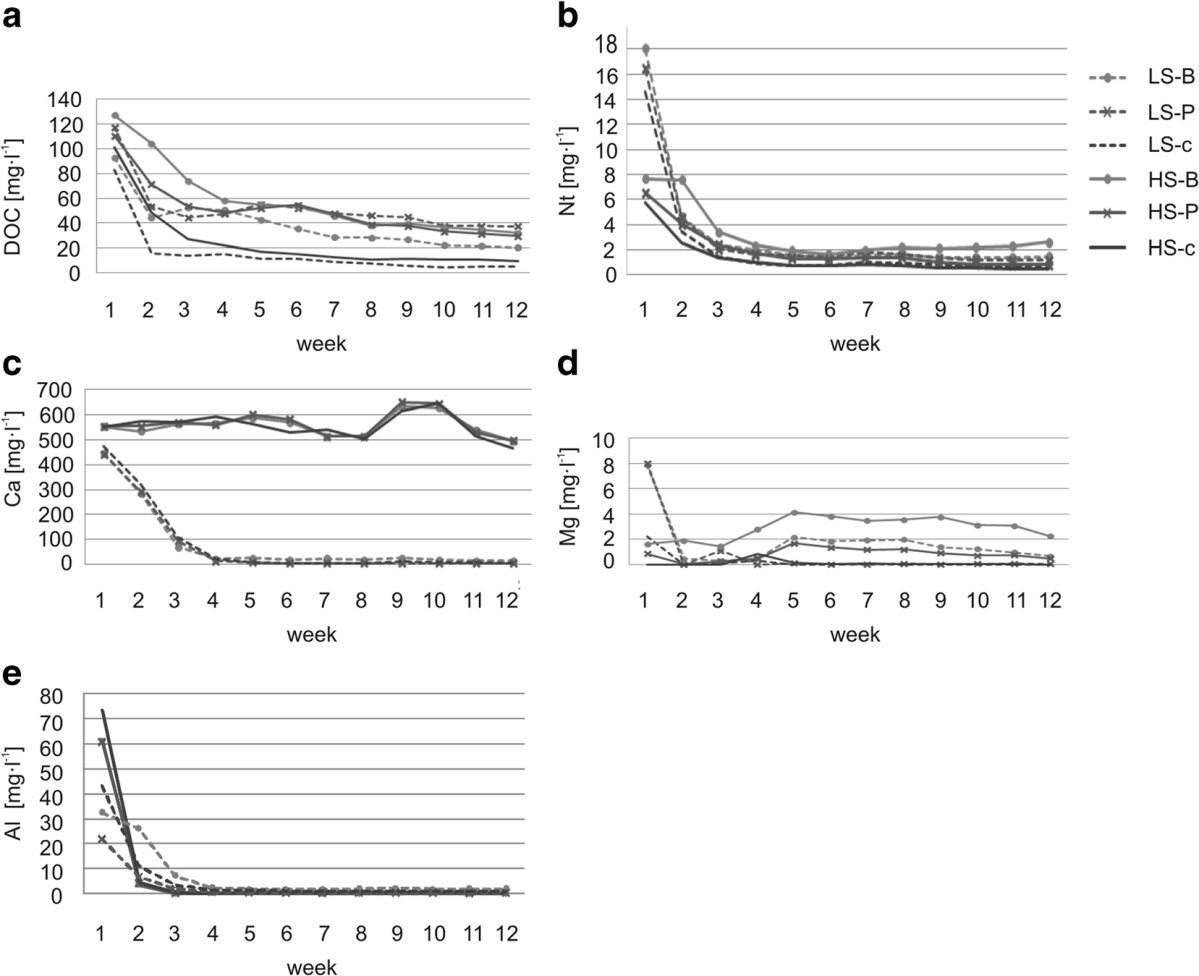
Changes in the DOC (**a**), Nt (**b**), Ca (**c**), Mg (**d**), and Al (**e**) content in solutions during the experiment under controlled conditions; abbreviations: LS soil substrate with low sulfur concentration, HS soil substrate with high sulfur concentration, B common birch litter, P Scots pine litter, c control samples with no litter addition

The most intensive leaching of DOC from composites containing both LS and HS was observed in the first 2 weeks. Later, the DOC concentrations in the solutions decreased (Fig. 7a). However, until the end of the experiment, higher DOC concentrations were in the litter containing composites than in substrates without litter addition (Table 3).

The highest leaching of Nt was observed in the first period of the experiment (up to week 2–3), wherein much more Nt was leached from the LS than from the HS substrates. After the fourth week, the Nt content stabilized at low Nt concentrations (0.43–2.58 mg l^−1^) (Fig. 7b). After 12 weeks of the experiment, significantly higher Nt leaching was observed in the HS substrate with birch litter (Table 3).

At the beginning of the experiment, Ca in HS solutions was considerably higher than Ca content in the LS solutions. During the experiment, Ca leaching from HS substrate composites was constantly high and only slightly diminished in week 12 (Fig. 7c). Ca leaching from the LS substrates was intensive in the first 3 weeks of the experiment and stabilized later on (Fig. 7c). In consequence, the Ca concentrations in leachates from LS composites at the end of the experiment were significantly lower than at the beginning of the experiment (Table 3).

At the beginning of the experiment, the Mg concentrations were much higher in leachates from LS substrates than from HS substrates. However, they dropped rapidly to very low values already in the second week and remained at this level till the end of the experiment (Fig. 7d). For the HS substrates, the Mg leaching was initially relatively low and subsequently peaked in week 4 and 5 and then gradually decreased toward the end of the experiment (Fig. 7d). Significantly higher than at the beginning were determined in composites with birch litter addition (Table 3). The Mg concentration in the solutions from LS substrate was significantly lower at the end of the experiment than at the beginning in each case wherein the highest Mg concentrations were in the leachates from composites with birch litter (Table 3).

The dynamics of Al leaching was similar in the LS and HS samples with the highest Al concentrations in the leachates taken in the first 2 weeks (Fig. 7e). In the first week, higher Al concentrations were measured in leachates from the LS substrates. Addition of litter tended to decrease the concentration of Al in the leachates compared with the control samples (Fig. 7e). This effect of litter addition was evident over the entire experiment for the LS substrates and up to the fourth week for the HS substrates.

## Discussion and Conclusions

Tree species litter affects soil properties by changing pH, nutrient content in the topsoil horizons, and the dynamics of organic carbon accumulation in soils (Hobbie et al. 2010; Vesterdal et al. 2008; Mueller et al. 2012). In forest habitats, especially on poor sandy soils sites, nutrients are mainly stored in the organic horizons (litterfall). Next, they are gradually released via mineralization processes (Baule and Fricker 1973). This process is important in providing nutrients to trees in oligotrophic condition, as a limited amount of nutrients in the soil can be replaced by the rapid biological cycling of elements from the organic horizon (Woś and Pietrzykowski 2015). Similarly as in natural forests, organic matter is important in tree nutrient balance in newly developed ecosystems on reclaimed and afforested post-mine sites characterized among others by lack of soil organic matter (Pietrzykowski and Krzaklewski 2007; Urbanová et al. 2014). Organic matter improves water retention and is a source of nutrients for plants and soil biota in the newly developed post-mine ecosystem (Frouz 2008).

The results indicate that the rate and amount of leached elements depended primarily on the amount of sulfur concentration in the substrate. After 12 weeks of the experiment, a significant reduction of sulfur content was observed in HS composites but no effect of litter addition was found. It was observed that despite significant reduction of St content in the HS composites, the degree of sulfur contamination was still very high. According to the values reported by Polish Institute of Crop and Soil Fertilization, it even exceeded by 35-fold very high sulfur content (heavy contamination), i.e., above 1000 mg kg^−1^ (Kabata-Pendias et al. 1995). Results in the LS variant indicate that pine litter had a significant impact on EC reduction and increased pH in the soil substrate. However, after 12 weeks of rinsing, the substrate pH (below 3.0) was still toxic to plants and most microorganisms. Low substrate pH may be related to litter decomposition when organic acids are released and a decrease of soil pH (Augusto et al. 2002). However, the main cause of acidification of substrates and soil solutions was high sulfur concentration. High sulfur concentrations in the soil are usually associated with acidification and low pH (Benison and Bowen 2013). Elemental sulfur application (at a rate of 300 μmol kg^−1^) decreases soil pH by 0.5 U (Kabata-Pendias 2011). The most common form of sulfur in soil is pyrite. Acid sulfate soils may arise from parent material containing pyrite associated with coal deposits, exposed to oxygen and undergoing pyrite oxidation that releases strong acid and residual sulfate (Rice and Herman 2012). In lignite mining areas, high sulfur content (above 0.1% S) in soils is usually connected with pyrites and marcasites (Katzur and Haubold-Rosar 1996; Pietsh 1996). Pyrite weathering increases rapidly, resulting in extremely low pH values amounting to 1.7–3.5 (Katzur and Liebner 1998).

The EC values in the initial substrates were high (1.87 mS cm^−1^ for the LS, 2.59 mS cm^−1^ for the HS substrates). Considering that the EC was measured at 5:1 (water/soil) ratio, the measured values corresponded roughly to 12 mS cm^−1^ for the LS and 16.5 mS cm^−1^ for the HS substrates measured in the soil paste (Shirokova et al. 2000). Such high EC values indicated high and very high salinity of the LS and HS substrates, respectively (Abrol et al. 1988). The load of salts in the LS substrates was relatively small as the EC values of the leachates decreased quickly during the experiment. At the end of the experiment, the EC values of the LS samples were low and corresponded to non-saline and low-salinity soils (Abrol et al. 1988). This was not the case for the HS substrates as the EC values of the leachates and the EC of the substrate at the end of the experiment were very high. The application of litter did not influence the salinity of the studied substrates.

Birch litter increased Nt leaching from the substrate with HS and Mg in LS and HS. At the beginning of the experiment, birch litter contained more Nt and Mg than pine litter. In the experiment involving rinsing substrates of technosols and litter application in controlled conditions, Woś and Pietrzykowski (2015) showed a significant impact of birch litter on the intensification of soil-forming processes and increased leaching of DOC, Nt, and Mg. It is obvious that application of organic matter, irrespective of its type, has a significant impact on the rate of DOC leaching. Menyailo et al. (2002) found that some species affected soil pH, DOC, and Mg but did not observe the impact of individual species on Ca and Nt content in soils. Also, Chodak and Niklińska (2010) found that the tree species had a significant impact on the chemical and microbial properties of mine soils, mainly through pH changes with SOC and Nt content.

Application of litter decreased Al leaching (in particular at the beginning of the experiment) thus contributed to lowering of Al toxicity. Aluminum is known to bind strongly to many organic compounds (Haynes and Mokolobate 2001) produced during litter decomposition, and this may explain lower Al leaching from the composite samples. Our results agree with previous studies which indicated that the addition of green manures and other organic amendments to acid soils reduced the concentration of Al in soil solution (Hue and Amien 1989; Wong et al. 1995). The total Al contents in the soil substrates decreased significantly after 12 weeks of leaching, wherein the composite samples tended to contain more Al than the control samples. This was apparently due to reduced Al solubility in litter-treated soils. Aluminum toxicity is associated with its soluble forms, and the total content of Al is not correlated with toxicity to soil biota (EPA 2003). Thus, higher total Al values in the composite samples at the end of the experiment suggest that litter addition reduced Al toxicity by transforming a part of soluble Al pool into insoluble forms.

The two applied types of litter differed in their basal respiration rate at the end of the experiment wherein pine litter exhibited higher values. Birch litter is known to contain more readily available C compounds (Kanerva et al. 2008) and is considered more degradable. Berg and Wessén (1984) reported that birch litter decomposed initially much faster than pine litter. We presume that readily available compounds from birch litter were rapidly metabolized at the beginning of the experiment resulting in lower RESP values after 12 weeks. A tendency for lower RESP values in the litter over HS substrates was probably due to their higher S content that by far exceeded values typical for S-contaminated soils (Kabata-Pendias et al. 1995).

Application of litter did not affect the respiration activity of the studied substrates as RESP values were extremely low both in the treated and control samples. The HS samples had much higher SOC content than LS samples; however, there was no difference in the RESP values between HS and LS. The factor analysis indicated that low pH was the main reason for the low microbial activity of the substrates. Although pH of the substrates increased over 12 weeks of the experiment, it was still within the range of toxic values and soil pH has been described as the major factor affecting soil microorganisms (Lauber et al. 2009; Rousk et al. 2010). Apparently, the load of base cations contained in the litter was not enough to neutralize toxic acidity of the studied substrates. To mitigate the negative effects of excessive sulfur concentration in soil associated with the sulfur industry, the lime (limestone, hydrated lime, quick lime) is recommended (Guidelines 1996; EPA 2007). McTee et al. (2017) reported that CaCO_3_ application was the most efficient way to improve ecological properties of acidic soils contaminated with elemental sulfur. We conclude therefore that the additional neutralization of toxic acidity would be required to enhance microbiological activity of the studied soil substrates.

The increase of St in pine litter and St, Nt, Ca, and Mg in birch litter is due to the fact that during decomposition of organic matter, its mass decreases while the concentration of these elements in organic matter increases. Elevated St content in the litter on LS and HS substrates may also be partly due to litter contamination with these materials during the experiment, e.g., by suction and soaking from the contaminated substrate (residual layer of the composite mineral). This may be one of the factors explaining an increase in element leaching from soil substrates through greater solubility (You et al. 1999; Kalbitz et al. 2000).

The results after 12 weeks of the experiment show that application of organic matter, especially in the case of soils most contaminated with sulfur, is not sufficient for fast detoxification. Probably in hotspots where the neutralization was not effective or inaccurate, it would be necessary to re-apply it, taking into account higher doses of flotation lime, as has been done so far in most areas of the former mine Jeziórko (Gołda 2003; Likus-Cieślik et al. 2017). In the next stage, it would be possible to introduce afforestation, which will gradually introduce sub-canopy litterfall and organic matter, which play a key role in soil-forming processes. Soil organic matter, even in its initial phase of accumulation, plays an important role in the nutrient balance in newly developed ecosystems on reclaimed and afforested post-mine sites (Pietrzykowski and Krzaklewski 2007; Urbanová et al. 2014). Decaying organic matter and biochemical processes produce more intensive leaching, and displacement of macronutrients into the soil profile and soil development will occur, but without neutralization, it will be a very-long-term process.

## Abbreviations

B: Common birch litter
P: Scots pine litter
LS: Soil substrate with low sulfur concentration
HS: Soil substrate with high sulfur concentration
c: Control samples (e.g., LS-c—a combination of soil substrate with low sulfur concentration with no litter addition)
s: Soil characteristics before the experiment
f: Soil characteristics at the end of the experiment—after 12 weeks (for substrate, litter and soil solution)
RESP: Basal respiration rate
